# Supplementary material for: Young adult-born neurons improve odor coding by mitral cells
Source: Nat Commun. 2020 Nov 17;11:5867. doi: 10.1038/s41467-020-19472-8 (PMC7673122; doi:10.1038/s41467-020-19472-8)
Supplement: Supplementary file 1 — Supplementary Information [file 41467_2020_19472_MOESM1_ESM.pdf]

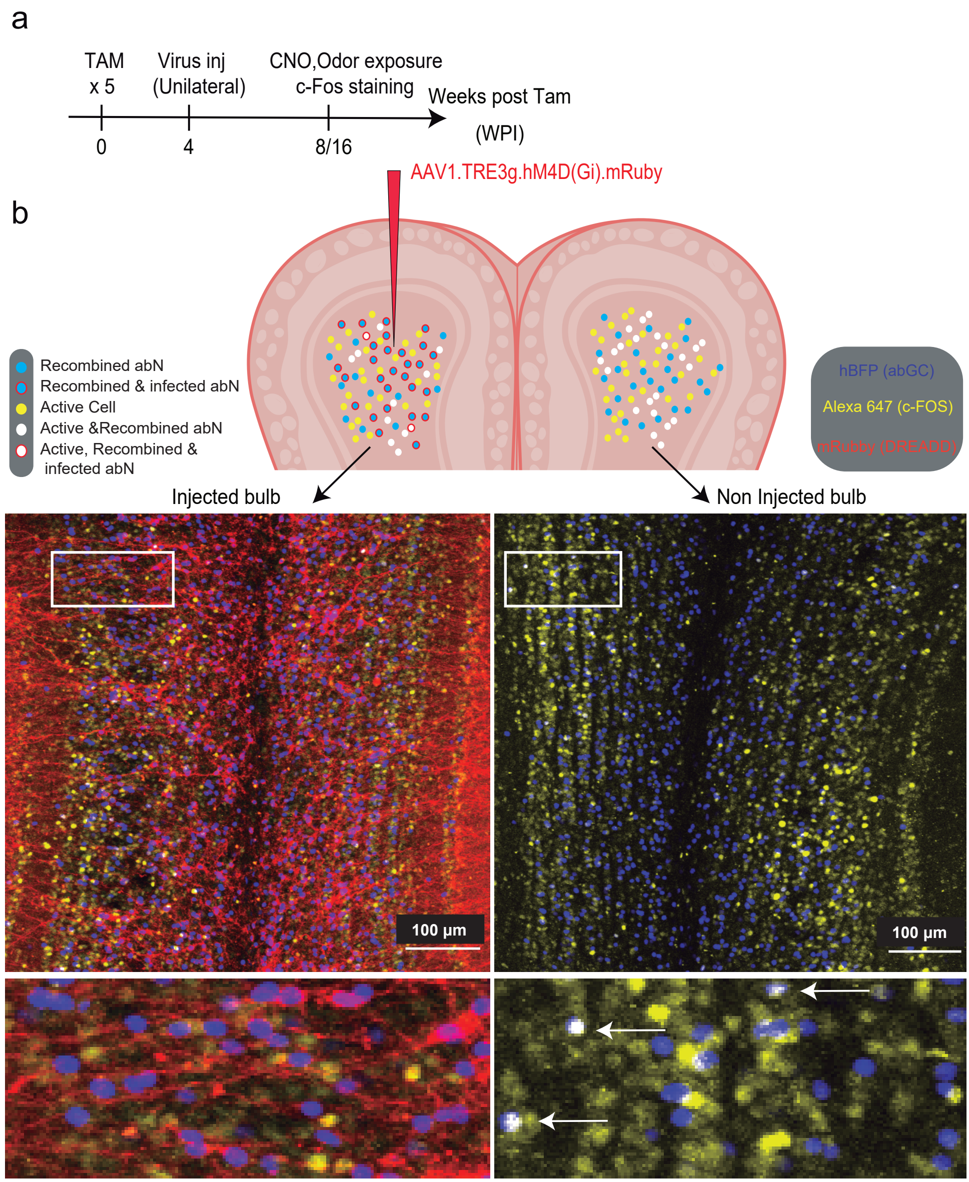

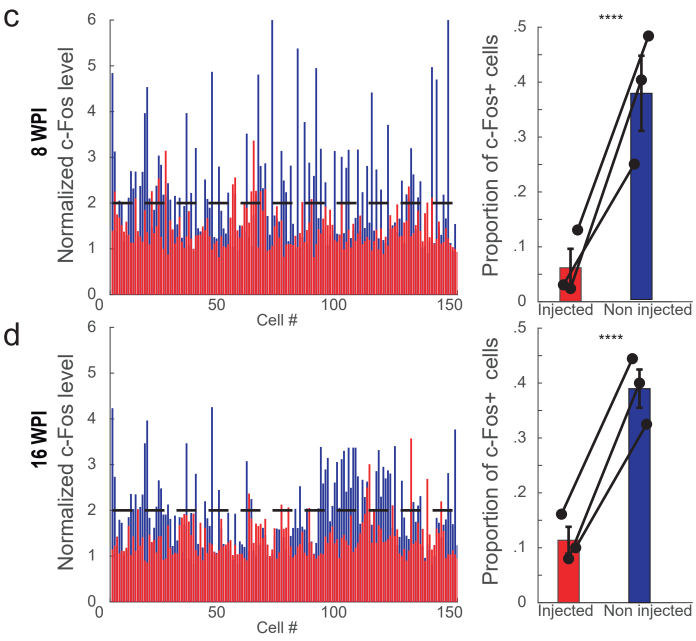

Supplementary Figure 1 – CNO administration effectively suppresses infected abGCs in different time points post injection. (**A**) Experimental timeline. (**B**) (Top) schematic illustration and (bottom) confocal micrographs of the injected (left) and non-injected (right) OBs of a mouse injected with CNO, stimulated with odors and assessed for c-Fos expression 2 hrs later (see Methods for details). Double labeling of abGCs and c-Fos is evident in the control (non-injected) hemisphere (arrows) but less so in the injected OB where abGCs were effectively silenced. Bottom - magnifications of white rectangular areas shown at the top. (**C**) (Left) c-Fos levels in abGCs from virus injected OB vs. the non-injected OB of 1 example mouse at 8WPI. Threshold was determined to be 2-fold higher from baseline level (dotted line). (Right) Quantification of the data for all mice (8WPI) exposed to odors and then sacrificed for histology. Proportion of c-Fos levels above threshold are higher in cells not infected by the DREAD virus. Single dots represent measurements from single mice (N=3 mice, *n*=400 DREADD+ and 400 DREADD- abGCs sampled from 12 slices, p<<.0001, binomial proportions test). (**D**) Same as C, but for mice 16WPI (N=3 mice, n=530 DREADD+ and 530 DREADD- abGCs sampled from 14 slices, p<<.0001, binomial proportions test). Statistical tests are two sided, and error bars represent the standard error of the mean (SEM). Panel b was created with BioRender.com.

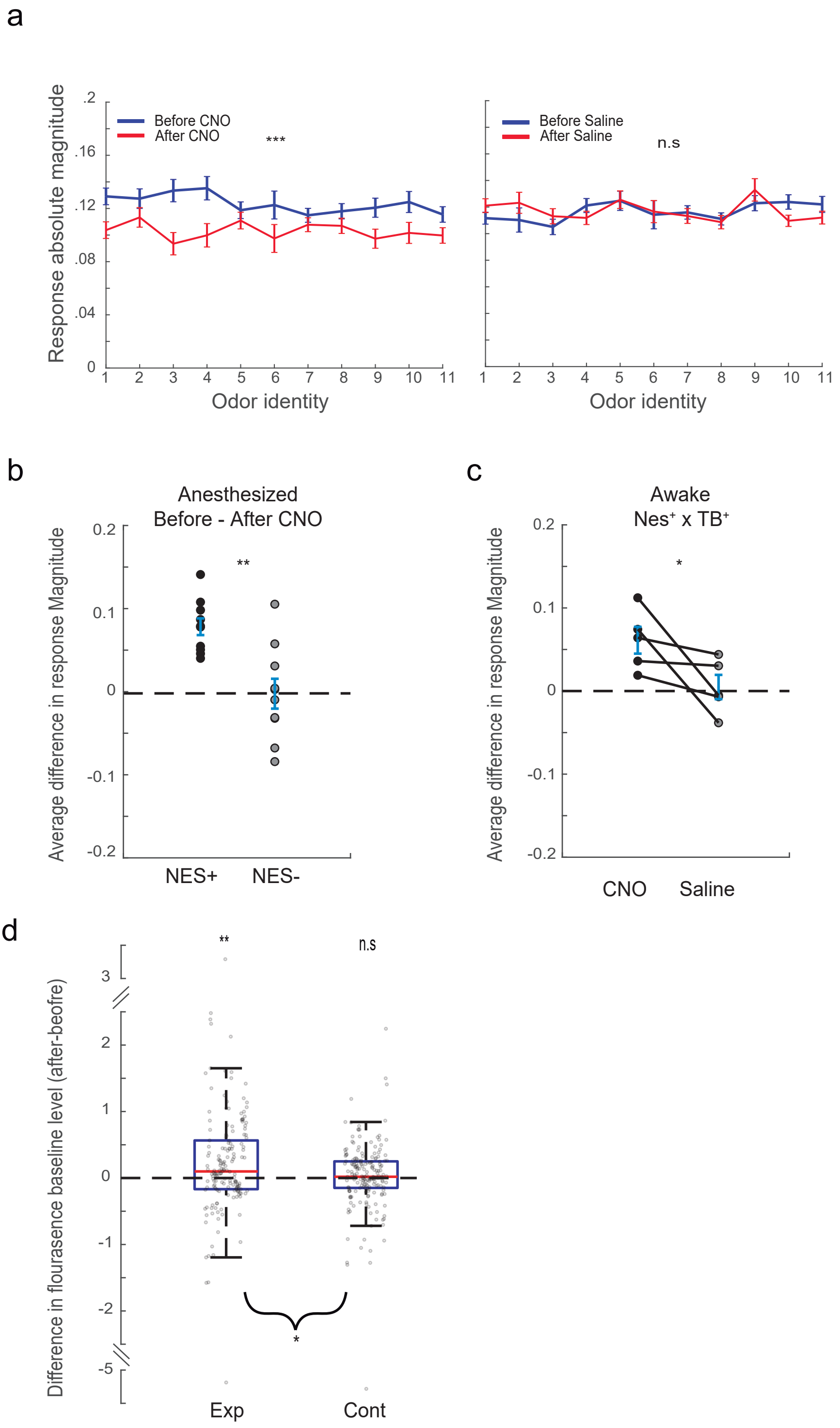

Supplementary Figure 2- Response magnitudes are suppressed across mice and odors and an estimate for spontaneous activity

(**A**) Left: Response magnitude per odor before (blue) and after (red) CNO. Right: before vs after saline. Data from awake NES+ experimental mice. (n=11 odors estimated as grand means over all mice before and after CNO\saline; CNO: p<.001; saline: p=0.12; Wilcoxon signed rank tests). (**B**) Difference in response magnitude following CNO injection in anesthetized mice, averaged per mouse (every data point is one mouse, N=20 mice, p<.01, Mann-Whitney U test). (**C**) Difference in response magnitude following CNO vs Saline injection in awake mice (N=10 recording sessions from 5 mice, p<.05, Wilcoxon signed rank test). (**D**) Fluorescence baseline before and after CNO in the absence of odor presentation in experimental and control groups. Baseline fluorescence was higher (i.e. after-before>0) after CNO in the experiment (Exp), but not in control (Cont) group. n_{exp}=170 cell-odor pairs, p<0.01, n_{cont}=194 cell-odor pairs, p=0.74, paired t-tests. The difference between the experimental and control groups was significant (p<0.05, unpaired t-test). Boxplots show the median (red), and lower/upper box bounds represent the 25th and 75th percentiles of the sample data, respectively. Whiskers represent boundsrepresent bounds for samples within +- 2.7 standard deviations (~99.5% of all data points). Statistical tests are two sided, and error bars represent the standard error of the mean (SEM), unless stated otherwise.

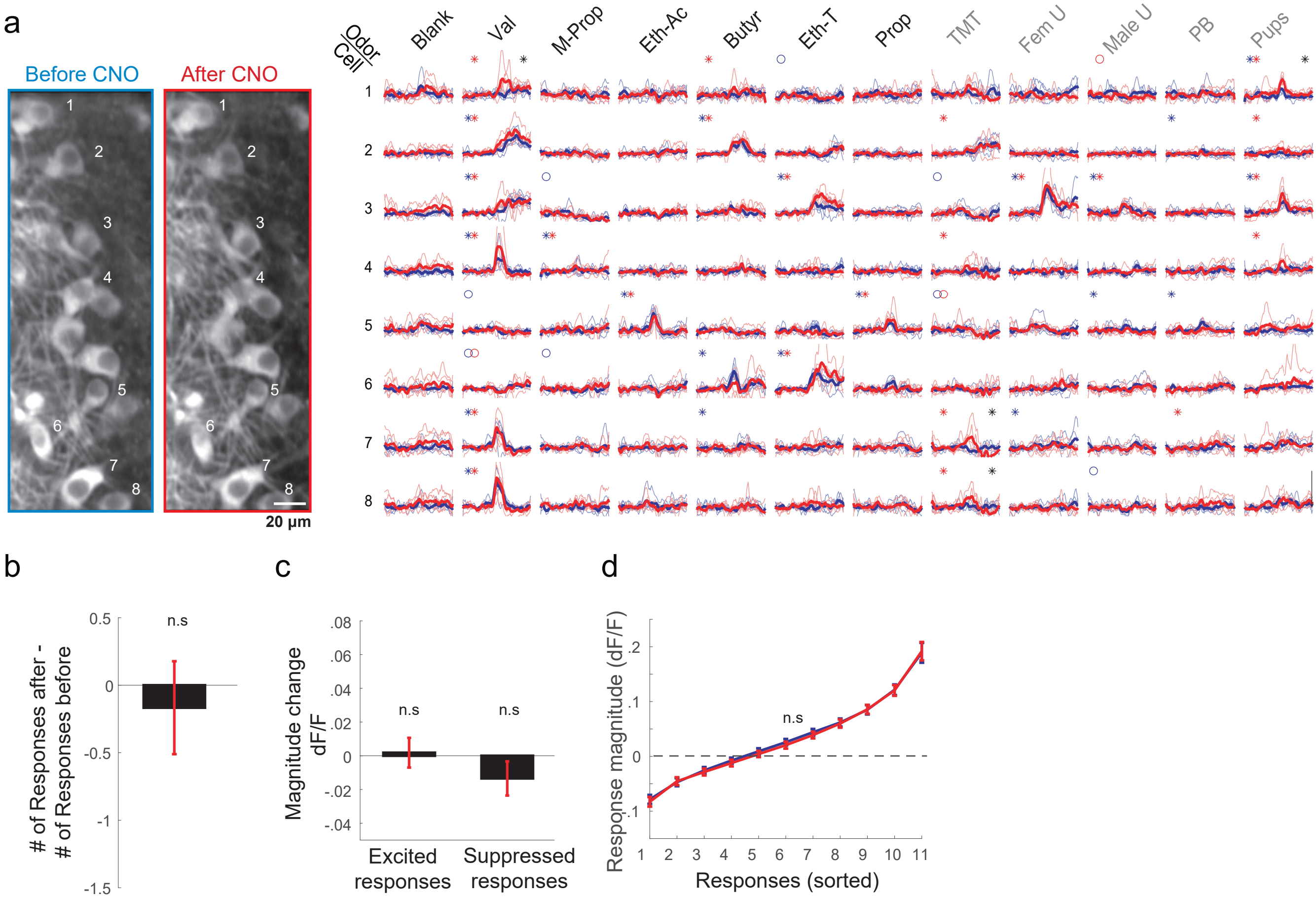

Supplementary Figure 3- CNO administration per se does not affect MCs odor evoked responses.

(**A**) Left- 2P micrograph of a representative field of MCs expressing GCamp6f before and after CNO administration. Right- Examples of calcium transients from 8 neurons (marked on the adjacent micrographs) in response to 6 monomolecular odors (black) and 5 natural odors (gray). Odor stimulation is denoted as a black horizontal line under each trace (2 sec). Thin traces are 5 single trials; thick traces are means. Blue/Red asterisks mark a statistically significant response for each condition. Black asterisks mark significant difference between the two conditions. Vertical Scale –100% dF/F. Odors are as described in Fig. 2E. (**B**) Change in responsiveness (number of responses) due to CNO injection at the absence of DREADD expression N=2 mice, n=42 cells, p=.63, one sample t-test). (**C**) The difference in absolute response magnitude due to CNO administration, at the absence of DREADD expression. Data shown separately for suppressed and excited responses.(Excited: n=159 cell-odor pairs, p=.84; Suppressed: n=70, p=.19; one sample t-tests). (**D**) Response magnitude in ranked order before (blue) and after (red) CNO administration, at the absence of DREADD expression. (n=42 cells, p=.77; Wilcoxon signed rank tests on cells curves' standard deviations before vs. after CNO). Statistical tests are two sided, and error bars represent the standard error of the mean (SEM), unless stated otherwise.

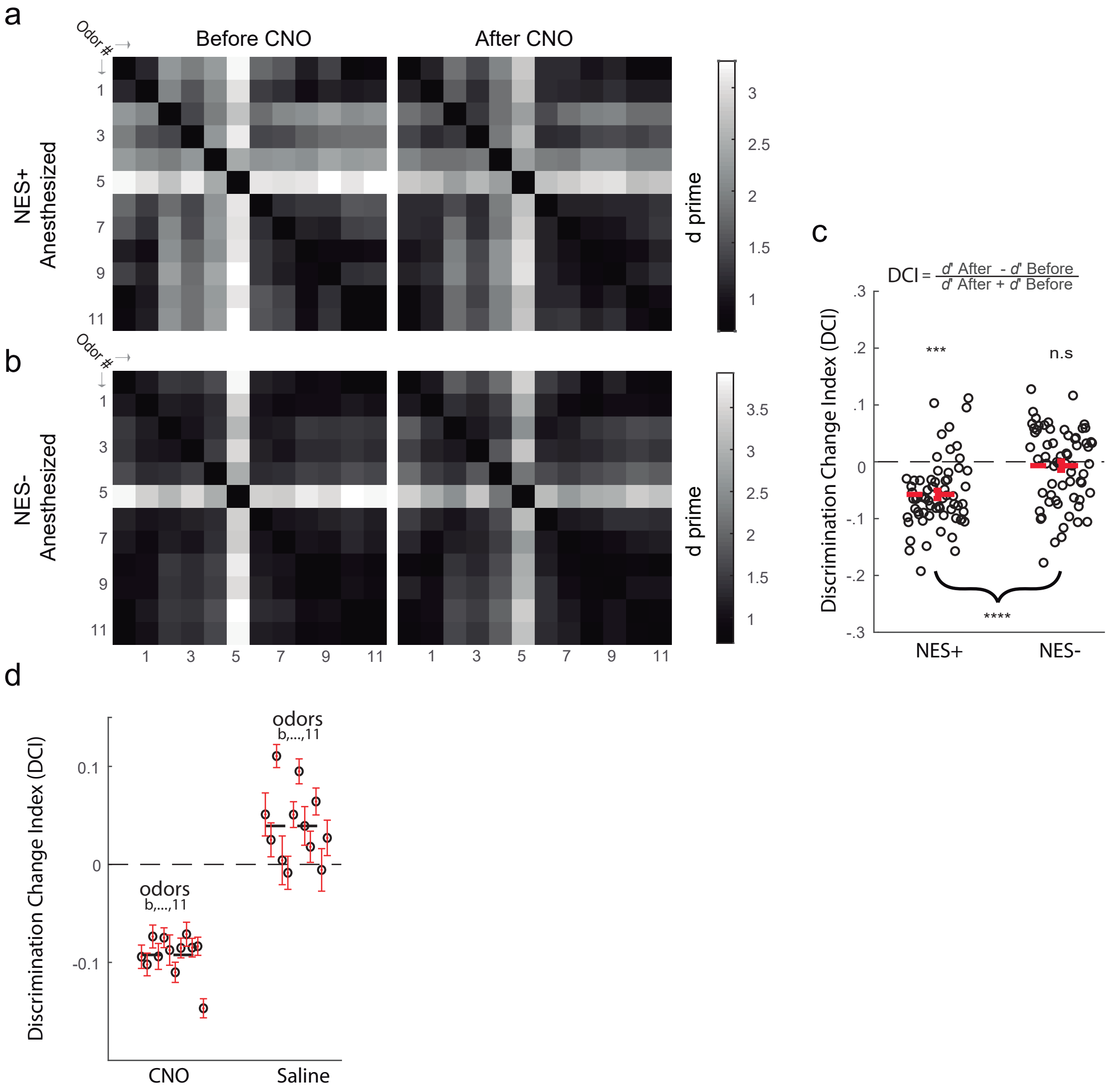

Supplementary Figure 4 – Population analysis - Supplementary results: general discrimination change in anesthetized mice and change per odor in awake mice.

(**A**) Matrices of d primes for all odor pairs, calculated in *n* dimensional space (*n*= number of cells) for each mouse and then averaged over all mice, before and after CNO. Data is from anesthetized mice (*N*=10). (**B**) Same as 'A', but for NES- mice (*N*=10). (**C**) Discrimination Change Index (DCI) following CNO injection in anesthetized mice (10 mice for each condition). (*N*= 66 DCIs for all comparisons; experiment vs control: $p<<.0001$, experiment vs 0: $p<.001$, control vs 0: $p=.41$, t-tests followed by Bonferroni correction). (**D**) DCI per odor following either CNO (*N*=5 mice) or Saline (*N*=5 mice) injection. Data is calculated from awake mice. Responses for all pairwise comparisons are presented in Fig. 4E. Statistical tests are two sided, and error bars represent the standard error of the mean (SEM).

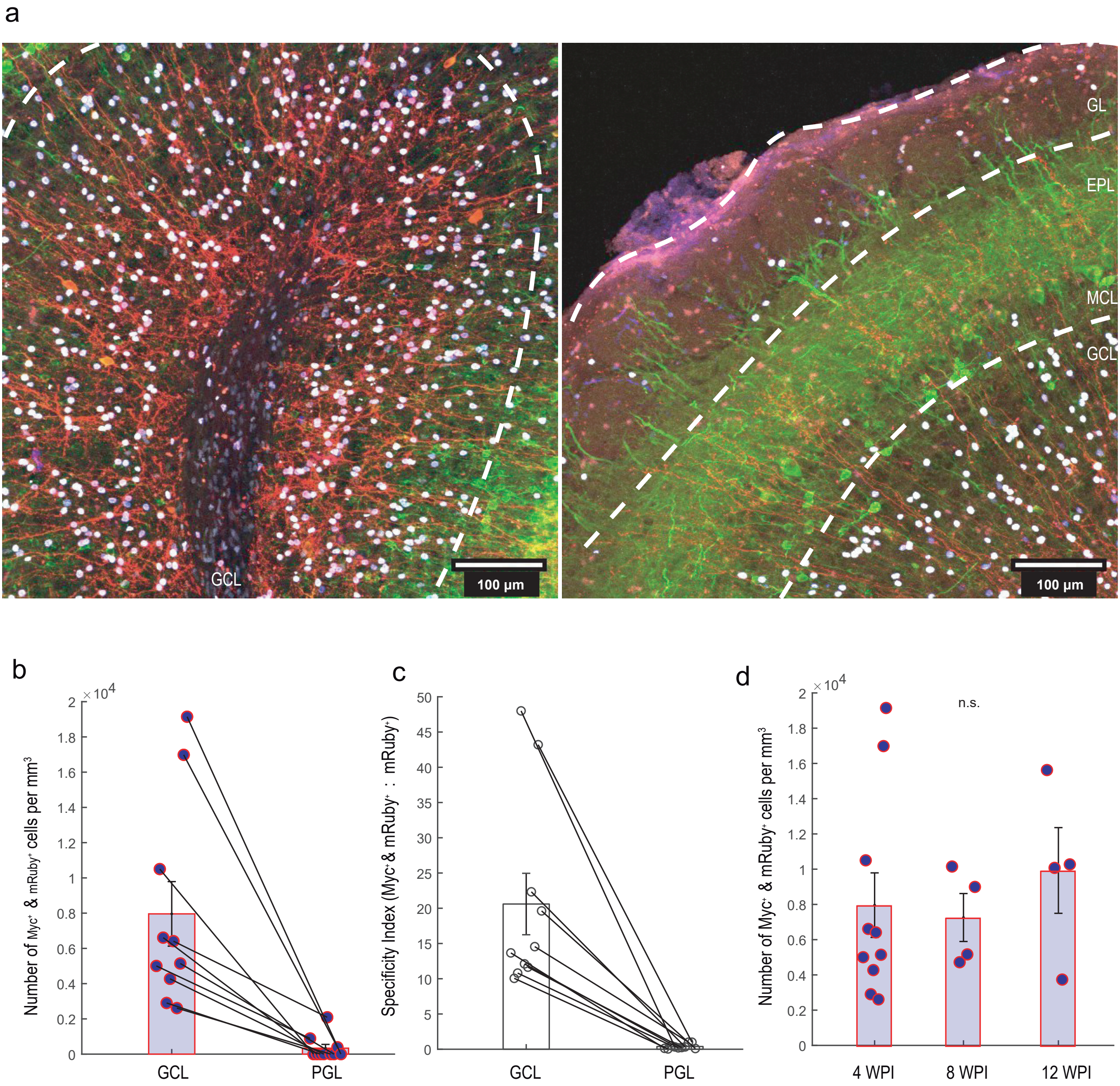

Supplementary Figure 5- Labelling efficiency and specificity in GCL vs. PGL and survival of abGCs in the GCL over time
(**A**) Confocal micrographs of the GCL (left) and PGL (right) post tamoxifen (16WPI) and virus injections (12WPI) show the abundance of DREADD infected (mRuby-red) abGCs (Myc\BFP- white) in the GCL compared to the PGL. Scale bars, 100 µm. PGL- Peri-glomerular layer, MCL- Mitral cells layer, GCL- Granule cell layer. (**B**) Quantification of double labelled cells (Myc+\BFP+ & mRuby+) in the PGL and GCL of all mice (*N*=10) shows significantly higher amounts in the GCL for all cases examined. Single dots represent measurements taken from individual mice. (**C**) Virus specificity was higher in the GCL compared to the PGL in all mice (*N*=10, see methods for definition). (**D**) Count of DREADD infected abGCs (mRuby+ & Myc+) 8, 12 and 16WPI. There are no significant differences in number of manipulatable cells over time (*N*=18 mice in total. $\chi^2_{2,15} = 0.56$. *p*=.75, Kruskal-Wallis test). Single dots represent measurements from single mice. Statistical tests are two sided, and error bars represent the standard error of the mean (SEM).

Supplementary Methods

Shani-Narkiss et al

SCHEMATIC MODEL

We model a network of Mitral cells (MCs) and Granule cells (GCs). We attempt to model the impact of adult-born Granule cells (abGCs) on the odor tuning properties of the network. The experimental observation is that silencing abGCs leads to a seemingly paradoxical effect, suppressing both inhibitory and excitatory odor-responses by MCs. We argue that this result can be explained quite simply by more "promiscuous" connectivity between MCs and abGCs together with overall elevated excitability of abGCs.

We model the three populations (MCs, GCs, and abGCs) as linear firing-rate neurons with distant-dependant connectivity, which for simplicity we take as having a Gaussian profile. We assume for simplicity that excitatory connections from the two GC populations onto MCs are identical, but we allow for broader MC input onto abGCs than onto GCs.

We assume that the odor input from OSNs is random and uncorrelated with the position of the neurons. For odor, k, neuron i receives input $I_0 + I_1 z_i^k$, where $\mathrm{Var}\left[z_i^k\right] = 1$. Thus the average input to MCs over all odors is I_0 and the standard deviation over odors is I_1.

The firing rate of neuron i in the MC population in response to odor k is:

$$r_i^{\mathrm{MC}}\left(\mathrm{odor}_k\right) = I_0 + I_1 z_i^k - \frac{1}{N_{\mathrm{GC}} + N_{\mathrm{abGC}}} \left(\sum_{j=1}^{N_{\mathrm{GC}}} J_{ij}^{\mathrm{MC} \leftarrow \mathrm{GC}} r_j^{\mathrm{GC}} + \sum_{j=1}^{N_{\mathrm{abGC}}} J_{ij}^{\mathrm{MC} \leftarrow \mathrm{abGC}} r_j^{\mathrm{abGC}} \right) \tag{1}$$

Meanwhile, the GC firing rates are given by:

$$r_i^{\mathrm{GC}} = \frac{1}{N_{MC}} \sum_{j=1}^{N_M} J_{ij}^{\mathrm{GC} \leftarrow \mathrm{MC}} r_j^{\mathrm{MC}} \tag{2}$$

$$r_i^{\mathrm{abGC}} = g \frac{1}{N_{MC}} \sum_{j=1}^{N_M} J_{ij}^{\mathrm{abGC} \leftarrow \mathrm{MC}} r_j^{\mathrm{MC}} \tag{3}$$

where $g > 1$ is the relative input-output gain of abGCs.

Defining Connectivity Profiles

We assume, for mathematical simplicity, that neurons in each population are distributed uniformly on a ring, such that we associate to each neuron j of population X an angle, $\theta_j^X = \frac{2\pi j}{N_X}$. We define the connectivity strength from a neuron in population Y to a neuron in a population X as a function of the angular distance between them as:

$$J_{ij}^{XY} = J_{XY} \exp\left(-\frac{\left(\theta_i - \theta_j\right)^2}{2\sigma_{XY}^2} \right) \tag{4}$$

where $X, Y \in \{\mathrm{MC}, \mathrm{GC}, \mathrm{abGC}\}$.

We assume that all spatial spreads are significantly smaller than the full extent of the population, such that the average connectivity from populations Y to X is:

$$J_{XY}^0 = \frac{J_{XY}\sigma_{XY}}{\sqrt{2\pi}} \tag{5}$$

For simplicity we assume that J_{XY} is identical for all pairs X, Y.

We assume the connectivity profiles differ only by the spatial spread of the input connectivity from MCs to GCs: $\sigma_{\text{abGC}\leftarrow\text{MC}} > \sigma_{\text{GC}\leftarrow\text{MC}}$:
So we parametrize the relative breadth of abGC connectivity as:

$$\sigma_{\text{abGC}-\text{MC}} = b\sigma_{\text{GC}-\text{MC}} \tag{6}$$

with $b > 1$.
So we have

$$J^0_{\text{abGC}-\text{MC}} = bJ^0_{\text{GC}-\text{MC}} \tag{7}$$

Reduced Model and Fixed Point Solution

Because of the assumption that z_i^k is independent of θ_i, we can assume that the effective input from population Y to neuron i in population X is dominated by the mean:

$$\frac{1}{N_Y}\sum_{j=1}^{N_Y} J_{ij}^{XY} r_j^Y \approx J^0_{XY}\bar{r}_Y \tag{8}$$

where $\bar{r}_Y$ is the average firing rate of population Y.

Thus the average firing rate for (ab)GCs is $J^0_{\text{(ab)GC}\leftarrow\text{MC}}\bar{r}_{\text{MC}}$.

Therefore the firing rate of MC i is approximated by

$$r_i^{\text{MC}}(\text{odor}_k) \approx I_0 + I_1 z_i^k - J^0_{\text{MC}\leftarrow\text{GC}}\left(J^0_{\text{GC}\leftarrow\text{MC}}(1-f) + gJ^0_{\text{abGC}\leftarrow\text{MC}}f\right)\bar{r}_M \tag{9}$$

where

$$f \equiv \frac{N_{\text{abGC}}}{N_{\text{abGC}} + N_{\text{GC}}} \tag{10}$$

is the proportion of abGCs.

We can rewrite this as

$$r_i^{\text{MC}} \approx I_0 + I_1 z_i^o - J_{\text{eff}}\left(1 - f + g_{\text{eff}} f\right)\bar{r}_{\text{MC}} \tag{11}$$

where we have written

$$J_{\text{eff}} \equiv J^0_{\text{MC}\leftarrow\text{GC}} \cdot J^0_{\text{GC}\leftarrow\text{MC}} \tag{12}$$

as the effective GC-mediated lateral inhibition of MCs to each other. And

$$g_{\text{eff}} = g \cdot b \tag{13}$$

as the overall effective excitability of abGCs relative to mature GCs. Note that we have reduced the model to a total of five parameters: Two parameters, I_0 and I_1, describing the external input onto MCs, and three parameters, J_{eff}, g_{eff} and f, describing the recurrent interactions between MCs and abGCs/GCs.

Now we can solve for $\bar{r}_M$, the average MC firing rate:

$$\bar{r}_{\text{MC}} = \frac{I_0}{1 + J_{\text{eff}}\left(1 + (g_{\text{eff}} - 1) f\right)} \tag{14}$$

And then we have for individual MC firing rates:

$$r_i^{\text{MC}}(\text{odor}_k) = \bar{r}_M + I_1 z_i^k \tag{15}$$

Extent of Tuning and Sharpening Due to abGCs

The extent of tuning is measured by taking the standard deviation over odors, divided by the mean, giving, before silencing abGCs:

$$\text{Tuning}_{\text{before}} = \frac{I_1}{\bar{r}_M} = \frac{I_1}{I_0}\left(1 + J_{eff}\left[1 + \left(g_{eff} - 1\right) f\right]\right) \tag{16}$$

When silencing abGCs we will have

$$r_{i,\,\text{after}}^{MC}\left(\text{odor}_k\right) \approx I_0 + I_1 z_i^o - J_{\text{eff}}\left(1 - f\right)\bar{r}_M \tag{17}$$

so

$$\bar{r}_{\text{MC, after}} = \frac{I_0}{1 + J_{\text{eff}}\left(1 - f\right)} \tag{18}$$

$$\text{Tuning}_{\text{after}} = \frac{I_1}{I_0}\left[1 + J_{\text{eff}}\left(1 - f\right)\right] \tag{19}$$

So that the impact of silencing abGCs on MC tuning is

$$\text{Sharpening}_{\text{abGCs}} = \frac{\text{Tuning}_{\text{before}}}{\text{Tuning}_{\text{after}}} = \frac{1 + J_{eff}\left[1 + \left(g_{\text{eff}} - 1\right) f\right]}{1 + J_{\text{eff}}\left(1 - f\right)} \tag{20}$$

CHERNOFF DISTANCE

We consider two odors, o_1 and o_2, and assume that the population of neurons fire a number of spikes, $\mathbf{n} = \{n_1 ... n_N\}$, which are independent Poisson distributions with means $\boldsymbol{\lambda}^{(1)}$ and $\boldsymbol{\lambda}^{(2)}$ respectively.

The Chernoff Distance between the populations responses to the two odors is defined by:

$$D_C\left(o_1, o_2\right) \equiv \max_{\alpha} D_{\alpha}\left(o_1, o_2\right) \tag{21}$$

where

$$D_{\alpha}\left(o_1, o_2\right) \equiv -\log\left[\underset{\mathbf{n}}{\text{Tr}} P^{\alpha}\left(\mathbf{n}|o_1\right) P^{1-\alpha}\left(\mathbf{n}|o_2\right)\right] \tag{22}$$

Independent, Poisson Variables

In the case where the population is independent this yields:

$$D_\alpha(o_1, o_2) = -\sum_{i=1}^{N} \log\left[\sum_{n_i=0}^{\infty} P^\alpha(n_i|o_1) P^{1-\alpha}(n_i|o_2)\right] \tag{23}$$

i.e. D_α *is extensive. Therefore we focus on the per-neuron* $D_\alpha^{(1)} = -\log\left[\sum_n P^\alpha(n|o_1) P^{1-\alpha}(n|o_2)\right]$*, for a single neuron.*

Now assume that $P(n|o_k) \sim \text{Poi}(\lambda_k)$ i.e. $P(n|o_k) = \frac{\lambda_k^n}{n!}\exp(-\lambda_k)$, then we have

$$D_\alpha^{(1)}(o_1, o_2) = -\log\left[\sum_n \frac{\lambda_1^{n\alpha}}{(n!)^\alpha}\exp(-\lambda_1\alpha)\frac{\lambda_2^{n(1-\alpha)}}{(n!)^{1-\alpha}}\exp(-\lambda_2(1-\alpha))\right] \tag{24}$$

$$D_\alpha^{(1)}(o_1, o_2) = -\log\left[\underbrace{\left(\sum_n \frac{\left(\lambda_1^\alpha\lambda_2^{1-\alpha}\right)^n}{n!}\exp\left(-\lambda_1^\alpha\lambda_2^{1-\alpha}\right)\right)}_{=1}\exp\left(\lambda_1^\alpha\lambda_2^{1-\alpha} - \lambda_1\alpha - \lambda_2(1-\alpha)\right)\right] \tag{25}$$

yielding

$$D_\alpha^{(1)}(o_1, o_2) = -\lambda_1^\alpha\lambda_2^{1-\alpha} + \lambda_1\alpha + \lambda_2(1-\alpha) \tag{26}$$

Thus the Chernoff Distance of the whole population is

$$D_C(o_1, o_2) = \max_\alpha \sum_{i=1}^{N} -\lambda_{1,i}^\alpha\lambda_{2,i}^{1-\alpha} + \lambda_{1,i}\alpha + \lambda_{2,i}(1-\alpha) \tag{27}$$

where $\lambda_{k,i}$ is the average spike count of the ith neuron in response to the kth odor.

In our setting, $\lambda_{k,i}^B = \frac{I_0}{1+J_{eff}(1+(g_{eff}-1)f)} + I_1 z_i^k$ before silencing and $\lambda_{k,i}^A = \frac{I_0}{1+J_{\text{eff}}(1-f)} + I_1 z_i^k$ after silencing. We will write

$$I_0^{\text{eff}} = \begin{cases} \frac{I_0}{1+J_{\text{eff}}(1+(g_{\text{eff}}-1)f)} & \text{Before Silencing} \\ \frac{I_0}{1+J_{\text{eff}}(1-f)} & \text{After Silencing} \end{cases} \tag{28}$$

These are defined by the random input tuning, z_i^k, and so we write the expected per-neuron D_α between the typical two odors as

$$D_\alpha^{(1)} = \mathbb{E}\left[-\left(I_0^{\text{eff}} + I_1 z_i^1\right)^\alpha \left(I_0^{\text{eff}} + I_1 z_i^2\right)^{(1-\alpha)} + \alpha\left(I_0^{\text{eff}} + I_1 z_i^1\right) + (1-\alpha)\left(I_0^{\text{eff}} + I_1 z_i^2\right)\right] \tag{29}$$

which simplifies to

$$D_\alpha = I_0^{\text{eff}} - \mathbb{E}\left[\left(I_0^{\text{eff}} + I_1 z\right)^\alpha\right]\mathbb{E}\left[\left(I_0^{\text{eff}} + I_1 z\right)^{(1-\alpha)}\right] \tag{30}$$

For small input tuning, $I_1 \ll I_0$, we write:

$$\mathbb{E}\left[\left(I_0^{\text{eff}} + I_1 z\right)^\alpha\right] \approx \left(I_0^{\text{eff}}\right)^\alpha - \frac{\alpha(1-\alpha)}{2}\frac{I_1^2}{\left(I_0^{\text{eff}}\right)^{2-\alpha}}\mathbb{E}\left[z^2\right] \tag{31}$$

We argue that by symmetry D_α must be maximized by $\alpha = 0.5$ (though this is straightforward also to prove), and so we have

$$D_C^{(1)} \approx I_0^{\text{eff}} - \left(\left(I_0^{\text{eff}}\right)^{0.5} - \frac{1}{8}\frac{I_1^2}{\left(I_0^{\text{eff}}\right)^{1.5}}\right)^2 = \frac{1}{4}\frac{I_1^2}{I_0^{\text{eff}}}\left(1 - \frac{1}{16}\left(\frac{I_1}{I_0^{\text{eff}}}\right)^2\right) \tag{32}$$

or to leading order:

$$D_C^{(1)\,\text{before}} \approx \frac{1}{4}\frac{I_1^2}{I_0}\left(1 + J_{\text{eff}}\left(1 + \left(g_{\text{eff}} - 1\right) f\right)\right) \tag{33}$$

$$D_C^{(1)\,\text{after}} \approx \frac{1}{4}\frac{I_1^2}{I_0}\left(1 + J_{\text{eff}}\left(1 - f\right)\right) \tag{34}$$

We see that to leading order D_C is inversely proportional to I_0^{eff}, and so we can compare Before and After:

$$\frac{D_C^{\text{Before}}}{D_C^{\text{After}}} \approx \frac{I_0^{\text{eff after}}}{I_0^{\text{eff before}}} = \frac{1 + J_{\text{eff}}\left(1 - f + g_{\text{eff}} f\right)}{1 + J_{\text{eff}}\left(1 - f\right)} \tag{35}$$

which is identical to the $\text{Sharpening}_{\text{abGCs}}$ in terms of the tuning curve.

MODEL PARAMETERS

First we note that our empirical data includes a count of the number of labeled abGCs. Based on existing estimates of overal GC density in the OB, we estimate that the monthly addition of abGCs as labeled in our experiments accounts for about 2.5% of GCs. Therefore we set $f = 0.025$, except where otherwise mentioned.

To interpret experimental data with our model we assume that calcium imaging measurement of $\frac{\Delta F}{F}$ for a given odor o_k, yields an estimate of the change in firing rate relative to baseline:

$$R_i\left(o_k\right) \equiv \frac{r_i\left(o_i^k\right) - \bar{r}}{\bar{r}} \tag{36}$$

Thus the standard deviation of the odor-evoked responses observed in the experiment yields a direct measure of the "Tuning" for each neuron, as defined above. We find

$$\text{Tuning}_{\text{before}-\text{empirical}} = 0.097 \pm .005 \tag{37}$$

$$\text{Tuning}_{\text{after}-\text{empirical}} = 0.075 \pm .004 \tag{38}$$

which are the mean and SEMs before and after silencing abGCs, respectively.

In our model this value depends on the input tuning $\frac{I_1}{I_0}$ to which we do not have empirical access, but as shown above, this factor drops out in the measure of $\text{Sharpening}_{\text{abGCs}}$,the ratio of $\text{Tuning}_{\text{before}}$ to $\text{Tuning}_{\text{after}}$. Thus any given value of $\text{Sharpening}_{\text{abGCs}}$ constrains the phase space of the two parameters, J_{eff}(the effective GC-mediated lateral inhibition) and g_{eff}(the product of abGCs input broadness and excitability relative to mature GCs) to a single (monotonically decreasing) curve. That curve is given by:

$$g_{\text{iso}-\text{Sharpening}}\left(J_{\text{eff}}\right) = \frac{\left(\text{Sharpening}_{\text{abGCs}} - 1\right)\left[1 + J_{\text{eff}}\left(1 - f\right)\right]}{J_{\text{eff}} f} \tag{39}$$

Therefore we use this empirical measure to identify a range of confidence for the parameter values, g_{eff}, J_{eff}.

We find the empirical average is

$$\text{Sharpening}_{\text{abGCs}-\text{Avg}} \equiv \frac{\text{Avg}\left[\text{Tuning}_{\text{before}-\text{empirical}}\right]}{\text{Avg}\left[\text{Tuning}_{\text{after}-\text{empirical}}\right]} \approx 1.31 \tag{40}$$

We define confidence borders on the value of $\text{Sharpening}_{\text{abGCs}}$ according to

$$\text{Sharpening}_{\text{abGCs}-\text{Upper}} \equiv \frac{\text{Avg}\left[\text{Tuning}_{\text{before}-\text{empirical}}\right] + \text{SEM}\left[\text{Tuning}_{\text{before}-\text{empirical}}\right]}{\text{Avg}\left[\text{Tuning}_{\text{after}-\text{empirical}}\right] - \text{SEM}\left[\text{Tuning}_{\text{after}-\text{empirical}}\right]} \approx 1.46 \tag{41}$$

$$\text{Sharpening}_{\text{abGCs-Lower}} \equiv \frac{\text{Avg}\left[\text{Tuning}_{\text{before-empirical}}\right] - \text{SEM}\left[\text{Tuning}_{\text{before-empirical}}\right]}{\text{Avg}\left[\text{Tuning}_{\text{after-empirical}}\right] + \text{SEM}\left[\text{Tuning}_{\text{after-empirical}}\right]} \approx 1.17 \tag{42}$$

The resulting range of parameters is displayed in Fig 6F. The solid white line is the curve $g_{\text{iso-Sharpening}}\left(J_{\text{eff}}\right)$ for $\text{Sharpening}_{\text{abGCs-Avg}}$, and the upper and lower dashed curves are those for $\text{Sharpening}_{\text{abGCs-Upper}}$ and $\text{Sharpening}_{\text{abGCs-Lower}}$, respectively.

In Fig 6G we explore the impact of changing the proportion of abGCs, and to that end we focus (somewhat arbitrarily) on the case $J_{\text{eff}} = 4$, and plot the analogous curves $f_{\text{iso-Sharpening}}\left(g_{\text{eff}}\right)$.
Having set these parameters in order to achieve the $\text{Sharpening}_{\text{abGCs}}$, we can now constrain the ratio of I_0 and I_1 by the observed Tuning values. For example, via

$$\frac{I_1}{I_0} = \frac{\text{Tuning}_{\text{after}}}{1 + J_{\text{eff}}\left(1 - f\right)} \tag{43}$$

In Fig 6E we set $J_{\text{eff}} = 4$ and this forces $g_{\text{eff}} = 15$. By construction, however, the tuning curve shown would be identical for any choice of $J_{eff} > 0$ together with the corresponding g_{eff} and $\frac{I_1}{I_0}$ as described. For our choice of parameters we have $\frac{I_1}{I_0} = 0.015$. Maximally according to the model, we have $\frac{I_1}{I_0} \approx \text{Tuning}_{\text{after}} = 0.08$, and for values of $J_{\text{eff}} \sim 15$ we have $\frac{I_1}{I_0} \sim 0.005$. In the limit of large J_{eff} we would have $\frac{I_1}{I_0} \propto \frac{1}{J_{\text{eff}}}$.

Deriving a numerical estimate for the Chernoff Distance requires an absolute scale. Firing rates of MCs in the literature are on the order of 20Hz spontaneous, and a single sniff cycle is on the order of 1s, so it may be appropriate to consider 20 spikes as our I_0^{eff}, which would make $I_0 \approx 80$, and therefore $I_1 \approx 1.2$ with a potential range of say, $[0.5, 5]$. We find

$$D_C^{(1)} \approx \begin{cases} 0.03 & \text{Before} \\ 0.02 & \text{After} \end{cases} \tag{44}$$

Assuming independence, the D_C of the population of N_{MC} neurons is $D_C = N_{\text{MC}} D_C^{(1)}$. In Fig 6H we plot

$$D_C = \frac{N_{\text{MC}}}{4} \frac{I_1^2}{I_0} \left(1 + J_{\text{eff}}\left(1 + \left(g_{\text{eff}} - 1\right) f\right)\right) \tag{45}$$

as a function of both N_{MC} and f, and mark the "iso-discrimination" curve, $N_{\text{iso-D}_\text{C}}\left(f\right)$, for D_C^{baseline} with $N_{MC}^{\text{baseline}} = 1000$ and $f^{\text{baseline}} = .025$ which yields $D_c \approx 30$. This curve is given by

$$N_{\text{iso-D}_\text{C}}\left(f\right) = N_{\text{MC}} \frac{1 + J_{\text{eff}}\left(1 + \left(g_{\text{eff}} - 1\right) f^{\text{baseline}}\right)}{1 + J_{\text{eff}}\left(1 + \left(g_{\text{eff}} - 1\right) f\right)} \tag{46}$$

In principle this curve depends on the particular choice of J_{eff} and g_{eff}. Nevertheless, as we show here, this dependence turns out to be very weak.

Near independence of the iso-discrimination curve to choice of parameters

To derive the dependence of $N_{\text{iso-D}_\text{C}}\left(f\right)$ on our choice of J_{eff} we substitute g_{eff} with $g_{\text{iso-Sharpening}}\left(J_{\text{eff}}\right)$ from above, which is a function of f^{baseline}. Importantly f^{baseline} is empirically estimated to be $f^{\text{baseline}} = 0.025 \ll 1$. Therefore (writing $S \equiv \text{Sharpening}_{\text{abGCs}}$):

$$g_{\text{iso-Sharpening}}\left(J_{\text{eff}}\right) \approx \frac{\left(S - 1\right)\left(1 + J_{\text{eff}}\right)}{f^{\text{baseline}} J_{\text{eff}}} \tag{47}$$

Plugging this into $N_{\text{iso-D}_\text{C}}\left(f\right)$ we find

$$N_{\text{iso-D}_\text{C}}\left(f\right) \approx N_{\text{MC}} \frac{S\left(1 + J_{\text{eff}}\right) f^{\text{baseline}}}{\left(S - 1\right)\left(1 + J_{\text{eff}}\right) f + \left[1 + J_{\text{eff}}\left(1 - f\right)\right] f^{\text{baseline}}} \tag{48}$$

We observe that for $ff^{\text{baseline}} \ll (S-1)\,f + f^{\text{baseline}}$ for the entire range of values of f and therefore we expand and simplify to find

$$N_{iso-D_C}\left(f \sim f^{baseline}\right) \approx N_{MC}\frac{Sf^{baseline}}{(S-1)\,f + f^{baseline}}\left(1+\frac{J_{eff}}{1+J_{eff}}\frac{ff^{baseline}}{(S-1)\,f+f^{baseline}}\right) \tag{49}$$

which shows that the influence of the one free parameter of our model, J_{eff}, on the iso-discrimination curve is as a second-order correction. This relative amplitude of this correction term is bounded by approximately $\frac{f^{\text{baseline}}}{S-1} \approx 0.06$. Thus resulting iso-discrimination curves are nearly quantitatively identical, independent of J_{eff}.

Supplementary Table 1- Details of statistical tests

Figure	Test used	Animals (*N*)	Units (*n*)	*P*-value	Degrees of Freedom (df) & **F/T/Z/R/U/Ks2stat/**χ^2
1e	Spearman R	3, 5 ,6, 4	Mice	*p*=3.8559e-08	*R*^2= 0.8357; b1 (slope, cells per day)= 297; df:16
2c	Mann-Whitney U	10,10	Mice	*p* =0.17	*U*=31.5; df: 18
2h	kstest2 (Kolmogorov-Smirnoff)	10,10	Cells. Exp=321; Cont=379 .	Exp: *p* =3.1281e-05 Cont: *p* =0.99	Exp: ks2stat =0.1838; Cont: ks2stat =0.0185
2j	Wilcoxon signed rank	10,10	Cells. Exp=321; Cont=379	Exp: *p*=7.3963e-10; Cont: *p*= 0.6036	Exp: z=6.1574; signed rank: 36087; df: 320; Cont: *Z*=0.5192 signed rank: 34897; df: 378
2k	Unpaired T-test	10,10	Cells. Exp=321; Cont=379	*p*=1.9291e-12	*T*= -7.1690; df: 698
2l	Unpaired T-test	10,10	Cell-odor pairs. 1258 Exp ,1558 Cont	*p* = 2.1314e-24	*T*= 10.2887 df: 2814
3c	kstest2 (Kolmogorov-Smirnoff)	5	Cells; Exp=170; Cont=152	Exp: *p* =7.6355e-05; Cont: *p* = 0.7134	ks2stat; Exp: =0.2412; Cont: =0.0789
3e	Wilcoxon signed rank test	5	Cells; Exp=170; Cont=152	Exp: *p*= 2.1853e-11 Cont: *p*=0.0709	Exp: *Z*=6.6931 rank=11569; df: 169; Cont: Z=-1.8063; rank=4832; df: 151
3f	Wilcoxon signed rank test	5	Cells; Exp=170; Cont=152	Exp: *p*= 3.7429e-06 Cont: *p* =0.8310	Exp: *Z*=4.6252 rank=10240; df:169; Cont: z=0.2134; rank= 5930; df: 151
3g	Un-paired t-test	5	Cells; Exp=170; Cont=152	*p* = 8.8062e-08	*T*= -5.4761; df: 320
3h	2 Un-paired t-tests	5	Cell-odor pairs. Excited: 531 Exp; 522 Cont; Supressed: 404 Exp; 251 Cont	Excited: *p* =1.0252e-6; Supressed: *p* =2.2932e-15	Excited: *T*= 4.9158; df: 1051 Suppressed: *T*= -8.1220; df: 653
4e	3 paired t-tests with Bonferroni correction	5	66 DCis. EXP (vs 0), Cont (vs 0), Exp vs Cont	Exp: *p* = 4.3304e-27 Cont: *p* = 1.1712e-05 Exp vs Cont; *p* = 9.0688e-21	Exp: *T*= -18.1086; df: 65; Cont: *T*= 4.7481 df: 65; Exp vs Cont *T*= 13.6573; df: 65
5c	Kstest2 (Kolmogorov-Smirnoff) two-tailed for 8WPI, one-tailed for 12,16WPI	8WPI: *N*=5; 12 WPI: *N*=4; 16WPI: *N*=4	Cells. 8WPI:113; 12WPI: 101; 16 WPI:104	1st: *p*=7.0691e-06; 2nd: *p* = 0.0357; 3rd: *p* = 0.9609	ks2stat 1st= 0.3274; 2nd=0.1782; 3rd=0.0192
5e	Chi-Square Test for equality of proportions	8WPI: *N*=5; 12 WPI: *N*=4; 16WPI: *N*=4	Cells-odor pairs. 8WPI:674; 12WPI:457; 16WPI:535	*p* =8.3778e-07	χ^2= 27.9850; df:2
5f	One-way ANOVA for repeated measures, followed by post-hoc Tukey-Kramer tests	8WPI: *N*=5; 12WPI: *N*=4; 16 WPI: *N*=4	Cells. 8WPI: 113; 12WPI: 101; 16WPI: 104	ANOVA; *p*=7.5796e-08; 8 vs 12; *p*= 0.0082862; 8 vs 16; *p*= 1.1571e-06; 12 vs 16; *p*= 0.0044005	ANOVA. *F*=17.965; df: 2, 182; 8 vs 12; df:100; 8 vs 16; df:103; 12 vs 16; df:91
5g	One-way ANOVA followed by post-hoc Tukey-Kramer tests	8WPI: *N*=5; 12 WPI: *N*=4; 16WPI: *N*=4	Excited Responses. 8WPI: 355 12WPI:300; 16 WPI:352	ANOVA; *p*=0.0033; 8 vs 12; *p*=0.1320; 8 vs 16 *p*= 0.0021; 12 vs 16 *p*=0.3902	ANOVA; *F*=5.75; df:2, 1004; 8 vs 12; df:653; 8 vs 16; df:705; 12 vs 16; df:650
5g	One-way ANOVA for repeated measures, followed by post-hoc Tukey-Kramer tests	8 WPI: *N*=5; 12WPI: *N*=4; 16WPI: *N*=4	Supressed Responses. 8 WPI: 292; 12WPI:144 16 WPI:158	ANOVA; *p*= 2.6984e-16; 8 vs 12; *p*=0.0002; 8 vs 16 *p*=~ 1.1291e-16 (t-test W/ Bonferroni correction); 12 vs 16; *p*=0.0003	ANOVA; *F*=38.11; df:2, 591; 8 vs 12; df:434; 8 vs 16; df:448; 12 vs 16; df:300
5h	One-way ANOVA followed by post-hoc Tukey-Kramer tests	8WPI: *N*=5; 12WPI: *N*=4; 16 WPI: *N*=4	66DCis/session. General test over the 3 sessions (*F*), then 8 vs 12, 8 vs 16, 12 vs 16	ANOVA; *p*= 4.5948e-31; 8 vs 12 *p*= 0.0002851; 8 vs 16; *p*= 9.5609e-10; 12 vs 16; *p*= 9.5609e-10	ANOVA; *F*=125.39, df:2,130 8 vs 12; 8 vs 16; 12 vs 16; for all df:65
Supp. 1c	Binomial proportions test	3	400 DREADD- cells, and 400 DREADD+ cells	*p*<<1e-20	χ^2= 112.047; df:1
Supp. 1d	Binomial proportions test	3	Cells. 530 DREADD-; 530DREADD+	*p*<<1e-20	χ^2= 104.0708; df:1
Supp. 2a	Wilcoxon signed rank tests	5	11 Odors' evoked responses	Exp: *p*= 9.7656e-04; Cont: *p*=0.1230	Exp: rank: 66; df:10; Cont: rank: 32; df:10
Supp. 2b	Mann-Whitney U-	10,10	Mice	*P*=0.0023	*Z*= 2.8347; ranksum: 143; df: 18
Supp. 2c	Wilcoxon signed rank. One-tailed.	5	Mice	*p*=0.0313	rank: 15 df:4
Supp. 2d	2 Wilcoxon signed rank tests	Exp: 5; Cont:7	Cells. Exp: 170; Cont: 194	Exp/Cont; *p* = 4.489e-04; *p* = 0.0779	Exp: z: 3.5095; rank: 9523; df: 169; Cont: z: 1.7630; rank: 10838; df: 193
Supp. 3b	One sample t-test (against 0)	2	42 Cells	*p*=0.6307	*T*= -0.4844; df: 41
Supp. 3c	2 t-tests, (against 0)	2	Excited responses deltas: n=159 Supressed responses deltas: n=70	Excited: p=0.8370; Supressed; *p*=0.1887	Excited: *T*= -0.2061; df: 158 Suppressed= 1.3276; df: 69
Supp. 3d	Wilcoxon signed rank test	2	42 Cells	*p*= 0.7689	*Z*=-0.2938; rank=428; df:41
Supp. 4c	3 t-tests with Bonferroni correction	10,10	66 DCis. EXP (vs 0), Cont (vs 0), Exp vs Cont	Exp: *p* = 4.2162e-11 Cont: *p* = 0.4095; Exp vs Cont (paired) *p* = 7.432e-05	Exp: *T*= 7.9101; df: 65; Cont:*T*= 0.8301; Exp vs Cont (paired); *T*= 4.2316; df:65 for all tests
Supp. 5d	Kruskal-Wallis test	10,4,4	Mice	*p*=0.7539	χ^2= 0.56; df:2,15
