# Supplementary material for: Young adult-born neurons improve odor coding by mitral cells
Source: Nat Commun. 2020 Nov 17;11:5867. doi: 10.1038/s41467-020-19472-8 (PMC7673122; doi:10.1038/s41467-020-19472-8)
Supplement: Supplementary file 3 — Reporting Summary [file 41467_2020_19472_MOESM3_ESM.pdf]

nature research

Corresponding author(s): Adi Mizrahi

Last updated by author(s): 2020 09 19

# Reporting Summary

Nature Research wishes to improve the reproducibility of the work that we publish. This form provides structure for consistency and transparency in reporting. For further information on Nature Research policies, see our Editorial Policies and the Editorial Policy Checklist.

## Statistics

For all statistical analyses, confirm that the following items are present in the figure legend, table legend, main text, or Methods section.

| n/a | Confirmed | |
|---|---|---|
| ☐ | ☒ | The exact sample size (*n*) for each experimental group/condition, given as a discrete number and unit of measurement |
| ☐ | ☒ | A statement on whether measurements were taken from distinct samples or whether the same sample was measured repeatedly |
| ☐ | ☒ | The statistical test(s) used AND whether they are one- or two-sided<br>*Only common tests should be described solely by name; describe more complex techniques in the Methods section.* |
| ☐ | ☒ | A description of all covariates tested |
| ☐ | ☒ | A description of any assumptions or corrections, such as tests of normality and adjustment for multiple comparisons |
| ☐ | ☒ | A full description of the statistical parameters including central tendency (e.g. means) or other basic estimates (e.g. regression coefficient) AND variation (e.g. standard deviation) or associated estimates of uncertainty (e.g. confidence intervals) |
| ☐ | ☒ | For null hypothesis testing, the test statistic (e.g. *F*, *t*, *r*) with confidence intervals, effect sizes, degrees of freedom and *P* value noted<br>*Give P values as exact values whenever suitable.* |
| ☒ | ☐ | For Bayesian analysis, information on the choice of priors and Markov chain Monte Carlo settings |
| ☒ | ☐ | For hierarchical and complex designs, identification of the appropriate level for tests and full reporting of outcomes |
| ☐ | ☒ | Estimates of effect sizes (e.g. Cohen's *d*, Pearson's *r*), indicating how they were calculated |

*Our web collection on statistics for biologists contains articles on many of the points above.*

## Software and code

Policy information about availability of computer code

| | |
|---|---|
| Data collection | Confocal microscopy was operated with Olympus FV 10-ASW 4.2. Fluorescence microscopy was operated with cellSens Dimension. 2-photon microscopy was operated with PrairieView software (version 5.5). Olfactometer was operated with RPmatrix and Matlab R2018b. |
| Data analysis | Image normalization for cell counting was done with Intensefy 3D (Yayon et al., 2018). Cell counting was done with Fiji (May 2017 release, 64-bit Windows version) 3D object counter. All data were analayzed in Matlab R2018b (Mathworks). |

For manuscripts utilizing custom algorithms or software that are central to the research but not yet described in published literature, software must be made available to editors and reviewers. We strongly encourage code deposition in a community repository (e.g. GitHub). See the Nature Research guidelines for submitting code & software for further information.

## Data

Policy information about availability of data

All manuscripts must include a data availability statement. This statement should provide the following information, where applicable:

- Accession codes, unique identifiers, or web links for publicly available datasets
- A list of figures that have associated raw data
- A description of any restrictions on data availability

All data and codes that support the findings of this study are available online with detailed instructions at https://github.com/MizrahiTeam/Shani-Narkiss-et-al.-2020..git

# Field-specific reporting

Please select the one below that is the best fit for your research. If you are not sure, read the appropriate sections before making your selection.

☒ Life sciences ☐ Behavioural & social sciences ☐ Ecological, evolutionary & environmental sciences

For a reference copy of the document with all sections, see nature.com/documents/nr-reporting-summary-flat.pdf

# Life sciences study design

All studies must disclose on these points even when the disclosure is negative.

| | |
|---|---|
| Sample size | A sample size of 10 mice was chosen a-priori for the physiology experiments in anesthetized mice, in accordance with the standard number of animals in system neuroscience studies, to allow a data set containing adequate number recorded cells (estimating ~ 40 cells recorded from each mouse). 10 mice were also taken for the control group, to achieve a sample of similar size. For the experiments conducted on awake mice, we chose a smaller sample size of 5 mice, since the implantation of chronic window is more complicated and requires ethic approval with higher severity level. The sample size for the anatomy/histology experiments was chosen similarly, such that the measured units (Cells, slices etc.) would be approximately equal between different groups and conditions. |
| Data exclusions | One mouse was excluded from the c-Fos estimation experiment, since its genotype was negative and no BFP+ cells were available for counting.<br>One mouse was excluded from the physiology experiment in anesthetized mice, since it died in the middle of recordings, and the data exhibited abnormal Ca+ activity. |
| Replication | Each mouse was an independent observation, enabling replication of the results across mice. All attempts of replication were successful. The number of replications varied between experiments according to the different amount of mice in each experiment, as reported. |
| Randomization | In some experiments, where possible, the same animal was used in both experiment and control groups (e.g. before-after CNO vs. before-after Saline, or DREADD in injected bulb vs. intact bulb of the same mouse). In other experiments, sibling animals were allocated to experiment\control group according to their positive\negative genotype, respectively. |
| Blinding | Investigators were blind to condition\group allocation whenever possible, as stated in the paper's methods section. Analyses were always conducted with either blindness to the group and/or any variable that may bias the results. Complete blinding was not possible in experiments where left bulb was injected and right bulb was used for control (supplementary Fig. 1). However, the counting of the cells in these tissues was done blindly to both the red channel (DREADD) and the far-red channel (c-Fos), thus allowing unbiased quantification in all cases. |

# Reporting for specific materials, systems and methods

We require information from authors about some types of materials, experimental systems and methods used in many studies. Here, indicate whether each material, system or method listed is relevant to your study. If you are not sure if a list item applies to your research, read the appropriate section before selecting a response.

## Materials & experimental systems

| n/a | Involved in the study | |
|---|---|---|
| ☐ | ☒ | Antibodies |
| ☒ | ☐ | Eukaryotic cell lines |
| ☒ | ☐ | Palaeontology and archaeology |
| ☐ | ☒ | Animals and other organisms |
| ☒ | ☐ | Human research participants |
| ☒ | ☐ | Clinical data |
| ☒ | ☐ | Dual use research of concern |

## Methods

| n/a | Involved in the study | |
|---|---|---|
| ☒ | ☐ | ChIP-seq |
| ☒ | ☐ | Flow cytometry |
| ☒ | ☐ | MRI-based neuroimaging |

## Antibodies

| | |
|---|---|
| Antibodies used | For staining, we used the following primary anti-bodies: a rabbit anti-Myc (abcam - ab9106), mouse anti-Myc (Santa Cruz, Cat #sc-40) or rabbit anti-Fos (Synaptic Systems, Cat #226003).These were followed by the following secondary antibodies: Alexa-647 conjugated Donkey anti-Rabbit , Alexa 647-conjugated goat anti-mouse-IgG or Alexa 647- conjugated goat anti-rabbit-IgG (Jackson ImmunoResearch). |
| Validation | All antibodies used in this paper are standard. Primary antibody procedures were validated by the procedure without primary AB incubation. |

## Animals and other organisms

Policy information about studies involving animals; ARRIVE guidelines recommended for reporting animal research

| | |
|---|---|
| Laboratory animals | Nestin-CreERT2 (Jax stock #016261, background strain C57BL/6, Lagace et al., 2007) were crossed to TB mice (Jax stock #031776, background strain FVB; Tasaka et al. 2018). Breeding colonies were obtained by coupling heterozygous Nestin-CreERT2 with homozygous TB breeders from both genders. All mice used for experiments were 7-9 weeks old females at the time of experiment initiation.<br>Temperature in animals housing was 21-24 Celsius degrees and 12/12 light-darkness regime was always maintained. |
| Wild animals | The study did not involve wild animals. |
| Field-collected samples | The study did not involve samples collected from the field. |
| Ethics oversight | All experimental procedures were approved by the Hebrew University Animal Care and Use Comitee |

Note that full information on the approval of the study protocol must also be provided in the manuscript.
